# Prevention of Mother-to-Child HIV Transmission Service Utilization among Pregnant Women in Northeast Ethiopia: A Cross-Sectional Survey

**DOI:** 10.1155/2020/7584975

**Published:** 2020-11-04

**Authors:** Betregiorgis Zegeye, Gorems Lemma, Abebe Balcha, Mitku Mammo Taderegew

**Affiliations:** ^1^Department of Public Health, College of Health Science, Debre Berhan University, Debre Berhan, Ethiopia; ^2^Department of Biomedical Sciences, College of Medicine and Health Sciences, Wolkite University, Wolkite, Ethiopia

## Abstract

**Background:**

Targeting pregnant women attending antenatal care clinics provides a unique opportunity for implementing the Prevention of Mother-to-Child Transmission (PMTCT) programs against human immunodeficiency virus (HIV) infection of newborn babies. The objective of this study was to assess the PMTCT service utilization rate and to characterize its reasons among pregnant women attending antenatal care clinics at selected public health facilities in Debre Berhan Town, Northern Ethiopia.

**Methods:**

A facility-based cross-sectional survey was conducted among 355 pregnant women from May 1 to June 15, 2019. The participants were selected by systematic random sampling technique, and data were collected using a pretested interviewer-administered structured questionnaire. Descriptive statistics like frequency, mean, and standard deviation were reported using text, table, and graphs.

**Results:**

The mean ages of the respondents were 24 (±5.6) years, and the majority of the respondents (287 (80.8%)) were urban residents. In this study, prevention of mother-to-child HIV transmission service utilization rate was 86.8%. The most frequently mentioned reasons for not utilization of services were fear of stigma and discrimination (42.6%), fear of rejection by partner (19.1%), fear of positive test results (17.0%), lack of awareness (12.7%), and wastage of time (8.5%). Hence, continuous health education and comprehensive counseling are necessary to increase the awareness and reduce stigma, fear of the positive result, and partner rejection.

## 1. Introduction

Mother-to-child transmission (MTCT) of human immunodeficiency virus (HIV) defined as the transmission of HIV from an HIV-positive mother to her baby at the time of pregnancy, labor, delivery, or breastfeeding [1, 2]. It is the most communal way that children become infected with HIV [1, 3]. Ninety-five percent of HIV-infected children acquired the infection through mother-to-child transmission during pregnancy, around the time of labor and delivery or during breastfeeding [4].

MTCT responsible for the vast majority of more than 700,000 estimated new HIV infections in children worldwide annually [5]. In 2015, there were approximately 2.1 million new HIV infections, 150,000 of which were among children. Most of these children were from sub-Saharan Africa, and the disease was acquired via their HIV-infected mothers at the time of pregnancy, delivery, or breastfeeding [6]. Despite the improvement made in the decline of MTCT in Ethiopia by enhancing the availability of HIV counseling and testing (HCT) services for pregnant women, low percentages of pregnant women receive the PMTC Services. A total of 400,000 mothers with reproductive age and 62,000 children aged 0 to 14 years with HIV-infection were reported in the country in the year 2016. Still, the percentage of pregnant women counseled and tested for PMTCT during an ANC visit or labor was 34% [7, 8].

The PMTCT plays a determinant role in minimizing the number of children being infected with HIV. Without any interference, 20–50% of infants would be infected with HIV; 5–10% during pregnancy, 10–20% during labor and delivery, and 5–20% through breastfeeding. By realizing the PMTCT program, the overall risk can be minimized to less than 2% [9].

Several influences lead to the low utilization of PMTCT services. Decreasing in the utilization of ANC, skilled birth attendants, and HIV counseling and testing (HCT) can affect the PMTCT service utilization [10]. Even if the number of health facilities offering PMTCT service has increased dramatically in Ethiopia, MTCT of HIV still persisting to be a challenge for the country may be due to high missed opportunities, dropout rates, and low utilization of services [11]. According to Ethiopia Demographic and Health Survey (EDHS) 2011 report, there is still a huge gap between the national ANC coverage (34%) and the percentage of women, who were counseled and tested and received the result during ANC (11%) [12]. Later on, the EDHS 2016 report showed that, nationally, the percentage of women who were counseled tested and received the result during ANC was 21.9% [8]. Particularly, in the Amhara region, the coverage indicates 31.5% [13].

To the best of our knowledge, there is little information on PMTCT service utilization and its associated factors in the study area. In spite of intensive efforts to scale-up PMTCT services in Ethiopia, the coverage and uptake of the service by the pregnant women remain low. Therefore, this study aimed to assess PMTCT service utilization rate and characterize its reasons among pregnant women following ANC at public health facilities of Debre Berhan Town, Northeast Ethiopia. The result of this study could provide up-to-date information about current level of utilization and its related reasons for low PMTCT services utilization. Such information renders evidence for applying PMTCT services and fills the gaps towards improving HIV prevention and control services, especially in the study area.

## 2. Methods and Materials

### 2.1. Study Area, Period, and Design

A facility-based cross-sectional survey was conducted in Debre Berhan Town, Northeast Ethiopia. Debre Berhan Town is located at about 130 km away from Addis Ababa, the capital city of Ethiopia. According to the 2015/16 annual statistical report of the town's Finance and Economic Development Office, the total population of the town was 57,787. There is one public referral hospital and one private primary hospital, 3 health centers, and 9 health posts. The study was conducted from May 1 to June 15, 2019.

### 2.2. Study Population and Sampling Techniques

The source populations were all pregnant women who attended ANC in the selected public health facilities during the study period and those pregnant women included in the study were considered as the study population. All pregnant women who attended the ANC clinic during the study periods were eligible for the study and those pregnant women who were severely ill and mentally and physically not capable of being interviewed were excluded from the study.

The sample size for this study was calculated using a single population proportion formula by considering a confidence level of 95%, marginal error 5%, 31% proportion of PMTCT utilization from a previous study [13], and 10% nonresponse rate. Hence, the final calculated sample size was 360.

**Figure 1:**
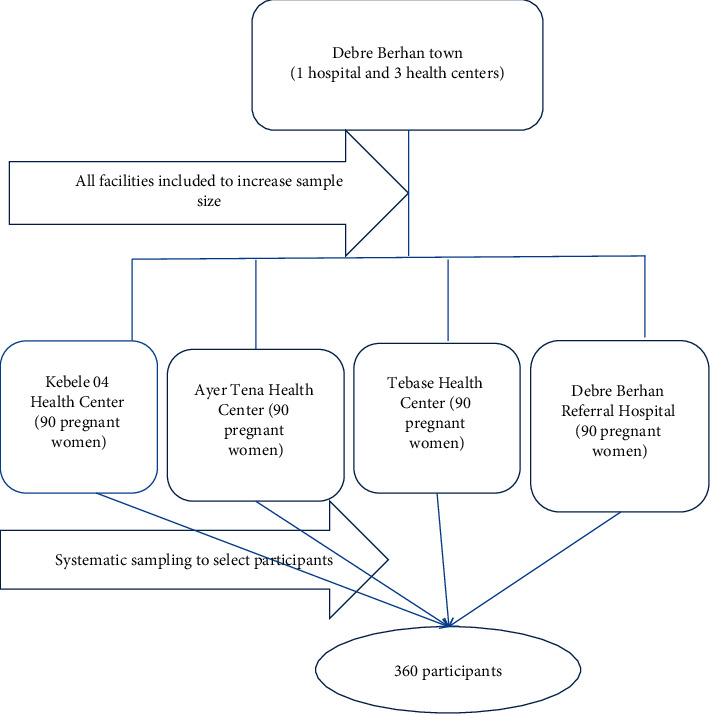
Schematic representation of sampling procedure for assessing PMTCT service utilization in Debre Berhan Town, June, 2019.

To select the study participants, first, we took all public health facilities (3 health centers and one referral hospital). The total sample was equally divided into those health facilities based on the assumption that ANC and PMTCT service utilization in all those health facilities were not significantly different from each other. Then, the average numbers of ANC visits per day in those health facilities were calculated as 10 as we have visited the ANC register of health facilities. Accordingly, about 200 pregnant women per health facility were expected to attend ANC during the data collection period (20 days). Then, a sampling interval of 2 was obtained by dividing the total expected number of pregnant women to the actual sample size. Thus, every second pregnant woman attending ANC clinics, irrespective of the numbers of previous ANC visits, was selected until the required sample size was achieved (Figure 1).

### 2.3. Data Collection Procedures

The data were collected using a pretested interviewer-administered structured questionnaire. The questionnaire was prepared by reviewing different published articles [1, 10, 11, 14, 15] with similar title in which the cultural and socioeconomic characteristics of study participants were similar to the target population of this study. The tool was primarily prepared in English language and translated to local language (Amharic) by an expert who had a good ability of the two languages and then another person with good ability of both languages retranslated back to English to ensure consistency. The internal validity of the questioner was also assessed by comparing questionnaire responses with objectives of the study during the pretest. The data were collected by trained midwives after taking two days of intensive training about the objectives of the study, tools, and ethical concerns.

The quality of the data was maintained by conducting a pretest on 5% of the sampled population in Keyit health Center, which was not part of the actual data collection area. Two-day training was given for data collectors and supervisors related to the objective of the study, tools, and ethical concerns. Moreover, the collected data were checked daily to safeguard its completeness.

The collected data were cleaned, coded, entered into EPI-info version-7 software, and exported to statistical package for social science (SPSS) version 20 for analysis. Descriptive statistics including frequencies, means, and standard deviations were computed to summarize the variables.

### 2.4. Ethics Approval and Consent to Participate

The study was conducted after ethical letters were obtained from Ethical Review committee of the Department of Public Health, Victory College, Debre Berhan Campus, Ethiopia. Then, permission was taken from hospital and health centers' clinical director offices. The data were collected after obtaining verbal and written informed consent from the study subjects. However, some study participants were under 18 years of age. Thus, consent to participate was collected from the partner, and also assent was obtained from those participants with age less than 18 years. To keep confidentiality, codes were used and unauthorized people did not have access to the data.

## 3. Results

### 3.1. Sociodemographic Characteristics of the Study Participants

A total of 360 pregnant women who attended the ANC visit were invited, and 355 of them were volunteered to participate in the study making the response rate 98.6%. The mean age of the respondents was 24 (±5.6) years, and the majority of the respondents (287 (80.8%)) were from urban areas. Out of the total respondents, 298 (83.9%) were orthodox christian by religion and 42 (11.8%) were muslims. The majority of the respondents (98.0%) were currently married. Nearly half of the respondents (174 (49.0%)) were housewives and 108 (30.4%) of their husbands were merchants by occupation. Regarding educational status, the majority 259 (72.9%) of the respondent's educational status was secondary and above, and fifty-eight (16.3%) of the respondents had no formal education (Table 1).

### 3.2. Obstetric Characteristics of the Respondents

Among the total of the respondents, 218 (61.4%) were multigravida and only 17 (7.8%) of them had no ANC visits in their last pregnancy. Of those respondents who gave birth previously, 186 (85.3%) were delivered at the health facilities. Nearly half of the respondents (51.0%) came with their partner and about 162 (45.6%) of the respondents discussed with their partner about HIV/AIDS. The majority (326 (91.8%)) respondents arrived at a health facility with less than 30 minutes. From all 308 HIV-tested pregnant women, the majority of them (96.1%) came with their partners for ANC follow-up visits. And, out of all the pregnant women who were not tested, most of them (85.1%) did not involve their partners (Table 2).

### 3.3. Knowledge about PMTCT

In this study, among all of the respondents, 330 (93.0%) respondents heard about MTCT of HIV. Three-hundred twenty (85.4%) participants knew PMTCT of HIV. Of these, 250 (70.4%) of the respondents knew ART drugs given for HIV-positive pregnant mothers could reduce the risk of HIV transmission (Table 3).

### 3.4. Utilization of PMTCT Services

Out of 355 respondents, 346 (97.5%) were precounseled and 308 (86.8%) were tested for HIV. Among respondents who were tested for HIV, 147 (47.7%) have been tested in the current visit, and the rest (161 (52.3%)) have been tested three months ago. Among those tested respondents, majority of them (303 (98.4%)) and two-hundred eighty-nine (95.4%) received their result and negative test results, respectively (Table 4).

### 3.5. Reasons for Not Utilizing PMTCT Services

**Figure 2:**
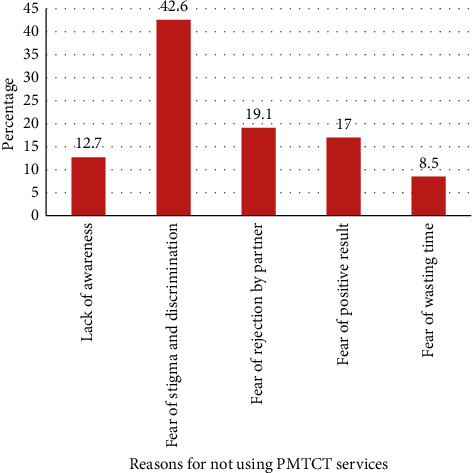
Reasons for not using PMTCT services among pregnant women attending ANC at public health facilities, Debre Berhan Town, June 2019.

Among 47 pregnant women who refused for HIV testing, nearly half of them (42.6%) were due to fear of stigma and discrimination followed by fear of rejection by partner (19.1%) (Figure 2).

## 4. Discussion

This study assessed the utilization of PMTCT services and the reasons for not using PMTCT services in selected public health facilities of Debre Berhan Town, Northeast Ethiopia. As part of ANC service, the study showed potential areas for the improvement of PMTCT service improvements. The primary sources of information about ANC for respondents in this study were health professionals including health extension workers. This could be partly because the majority of the respondents were from urban areas and had access to health facilities with less than 30 minutes of travel.

In the present study, the PMTCT service utilization among ANC attendants was found to be 86.8%. This finding was lower as compared to that of a previous study conducted in Addis Ababa, central Ethiopia, which showed 94% of pregnant women accepted HIV counseling and testing [10]. This could be because more respondents of our study who visited the health facilities came from rural areas as compared to the respondents who were in Addis Ababa. Utilization of PMTCT service in this study is similar to a study conducted in Sebeta, Central Ethiopia [11] which indicated that 86.9% of the respondents reported that they had been tested for HIV during the study periods either in the selected health facility or elsewhere. Moreover, our finding was nearly similar to a study conducted in Oromia regional state, Ethiopia, in 2016 which showed a service utilization rate of 83% [1]. However, the result of our study is higher than that of the EDHS 2016 report in Amhara regional state of Ethiopia, which indicated that only 31.5% of the respondents were counseled and tested and received results. This might be because most respondents in this study were urban residents, and there might be some service improvement as well as an increase in the level of awareness through time [8].

This study also assessed the reasons for not utilizing the PMTCT services. Fear of stigma and discrimination, fear of rejection by a partner, fear of positive result, lack of awareness, and wastage of time were the most common reasons reported by the study participants for refusals of PMTCT service utilization. In line with our findings, a study conducted in Sebeta showed that waiting for a long time for the service and lack of awareness and knowledge about the MTCT and PMTCT service utilization were the commonly mentioned reasons [1].

This study also agreed with the study in Addis Ababa, indicating that the lack of awareness about the availability and benefits of ANC/PMTCT services, shortage of PMTCT service providers, lack of adequate and separate room for PMTCT services, poor involvement of partners/husbands in ANC/PMTCT services, poor disclosure of HIV-status to partners, and psychological unpreparedness due to fear of being positive for HIV were the main barriers preventing the mothers from HIV testing [7].

Due to descriptive nature of study design used, it was not possible to correlate variables and to determine the cause and effect relationships which was the limitation of the study.

## 5. Conclusions

In this study, the PMTCT service utilization among ANC attendees was found to be 86.8%. This indicates that utilization of PMTCT services was low compared to the national direction of providing the services to all ANC attendants. The most frequently mentioned reasons for no utilization of PMTCT services were fear of stigma and discrimination followed by fear of rejection by the partners. It is very important to strengthen the health information provision about PMTCT services and broaden the channels of communication to reach a wider audience for conveying the needed information to decrease stigma and discrimination in the community. In addition, the involvement of partners is needed, and providing comprehensive couple counseling is also required to avoid result-related fear and to reduce partner rejection fears.

## Figures and Tables

**Table 1:** Sociodemographic characteristics of pregnant women attending ANC at public health facilities, Debre Berhan Town, June 2019 (*N* = 355).

Variable	Category	Frequency (*N* = 335)	Percent
Age (years)	15–24	201	56.6
	25–34	98	27.6
	≥35	56	15.8

Ethnicity	Amhara	278	78.3
	Oromo	57	16.1
	Gurage	14	3.9
	Tigre	6	1.7

Religion	Orthodox christian	298	83.9
	Muslim	42	11.8
	Protestant and others	15	4.3

Educational status	No formal education	58	16.3
	Elementary	38	10.7
	Secondary school	61	17.2
	Diploma and above	198	55.7

Occupational status	Professional	18	5.1
	Semiprofessional	39	11.0
	Skilled	82	23.1
	Semiskilled	88	24.7
	Manual	128	36.1

Current marital status	Married	348	98.0
	Divorced	7	2.0

Resident	Urban	287	80.8
	Rural	68	19.2

Partner's occupation	Farmer	27	7.6
	Government employee	19	5.4
	Merchant	108	30.4
	Other	201	56.6

**Table 2:** Obstetrics and other factors among pregnant women attending ANC at public health facilities, Debre Berhan Town, June 2019 (*N* = 355).

Variable	Category	Frequency	Percent
Time taken to arrive at the health facility	<30 min	326	91.8
	30–1 hr	22	6.2
	>1 hr	7	2.0

Number of pregnancies	1	137	38.6
	2	56	15.8
	3	71	20.0
	≥4	91	25.6

ANC in last pregnancy	No	17	7.8
	Yes	201	92.2

Place of last delivery	Home	10	4.9
	HF	186	88.5
	Others	14	6.6

Number of ANC visits in the current pregnancy	One	72	20.3
	Two	78	22.0
	Three	149	42.0
	Four and above	56	15.8

Partner involvement in ANC	No	174	49.0
	Yes	181	51.0

Discussion with a partner about HIV	No	162	45.6
	Yes	193	54.4

*Note.* ANC: antenatal care; HIV: human immunodeficiency virus.

**Table 3:** Knowledge-related factors among pregnant women attending ANC at public health facilities, Debre Berhan Town, June 2019 (*N* = 355).

Variable	Category	Frequency	Percent
Heard about MTCT of HIV	No	25	7.0
	Yes	330	93.0

Source of information	Social/mass media	90	25.4
	Health professional	242	68.2
	Others	23	6.4

HIV can be transmitted during pregnancy	No	42	11.8
	Yes	309	87.0
	I do not know	4	1.1

HIV can transmitted during labor	No	40	11.3
	Yes	298	83.9
	Do not know	16	4.5

HIV can transmitted during lactation	No	45	12.6
	Yes	293	82.5
	Do not know	17	4.9

ANC important for PMTCT	No	22	6.2
	Yes	317	89.3
	Do not know	16	4.5

Health facility delivery important for PMTCT	No	24	6.8
	Yes	314	88.5
	Do not know	17	4.8

Breastfeeding counseling important for PMTCT	No	35	9.9
	Yes	312	87.9
	Do not know	8	2.3

HIV test done at HC/hospital	No	21	5.9
	Yes	334	94.1

ART can reduce MTCT of HIV	No	48	13.5
	Yes	250	70.4
	I do not know	57	16.1

**Table 4:** HIV testing and counseling as PMTCT service utilization among pregnant women attending ANC at public health facilities, Debre Berhan Town, June 2018 (*n* = 355).

Variable	Category	Frequency	Percent
Have got precounseling	No	9	2.5
	Yes	346	97.5

Tested HIV	No	47	13.2
	Yes	308	86.8

Received test result	No	5	1.6
	Yes	303	98.4

Result of the test	Positive	3	1.0
	Negative	289	95.4
	Do not know	11	3.6

Have got posttest counseling	No	5	1.6
	Yes	303	98.4

Linked to ART	No	0	0
	Yes	3	100

Readiness to go ART clinic right now	No	0	0
	Yes	3	100

Satisfaction by service	No	86	24.2
	Yes	269	75.5

## Data Availability

The datasets used and/or analyzed during this study are available from the corresponding author on reasonable request.

## References

[1] Feyera A., Megerssa B. E., Legesse D., Hailemichael F. A. (2017). Prevention of mother to child transmission of HIV/AIDS: service utilization and associated factors among selected public health facilities in Ethiopia. *Medical Practice and Reviews*.

[2] Tolle M. A., Dewey D. (2010). Prevention of mother-to-child transmission of HIV infection. *HIV Curriculum for the Health Professional*.

[3] World Health Organization (2011). *Global HIV/AIDS Response: Epidemic Update and Health Sector Progress towards Universal Access: Progress Report*.

[4] Global A. *Information and Education on HIV and AIDS. Prevention of Mother-to-Child Transmission (PMTCT) of HIV*.

[5] Federal Democratic Republic of Ethiopia (2014). Country Progress Report on the HIV Response. https://www.unaids.org/sites/default/files/country/documents/ETH_narrative_report_2014.pdf.

[6] World Health Organization (2009). *PMTCT Strategic Vision 2010-2015: Preventing Mother-To-Child Transmission of HIV to Reach the UNGASS and Millennium Development Goals: Moving towards the Elimination of Paediatric HIV*.

[7] World Health Organization (2016). HIV Country profile Ethiopia 2016. https://www.who.int/hiv/data/Country_profile_Ethiopia.pdf?ua=1.

[8] Central Statistical Agency (CSA) (Ethiopia) (2016). *Ethiopia Demographic and Health Survey 2016*.

[9] World Health Organization (2007). *Guidance on Global Scale-Up of the Prevention of Mother-Child Transmission of HIV: Towards Universal Access for Women, Infants and Young Children and Eliminating HIV and AIDS among Children*.

[10] Deressa W., Seme A., Asefa A., Teshome G., Enqusellassie F. (2014). Utilization of PMTCT services and associated factors among pregnant women attending antenatal clinics in Addis Ababa, Ethiopia. *BMC Pregnancy and Childbirth*.

[11] Merga H., Woldemichael K., Dube L. (2016). Utilization of prevention of mother-to-child transmission of HIV services and associated factors among antenatal care attending Mothers in Sebeta town, Central Ethiopia. *Advances in Public Health*.

[12] Tarekegn S. M., Lieberman L. S., Giedraitis V. (2014). Determinants of maternal health service utilization in Ethiopia: analysis of the 2011 Ethiopian Demographic and Health Survey. *BMC Pregnancy and Childbirth*.

[13] Federal Democratic Republic of Ethiopia Ministry of Health. Health and Health Related Indicators, 2016, http://repository.iifphc.org/bitstream/handle/123456789/395/Health%20and%20Health%20Related%20Indicator%202017.pdf?sequence=1&isAllowed=y

[14] Bissek A. Z., Yakana I. E., Monebenimp F. (2011). Knowledge of pregnant women on mother-to-child transmission of HIV in Yaoundé. *The Open AIDS Journal*.

[15] Demissie A., Deribew A., Abera M. (2009). Determinants of acceptance of voluntary HIV testing among antenatal clinic attendees at Dil Chora Hospital, Dire Dawa, East Ethiopia. *Ethiopian Journal of Health Development*.

